# Evaluation of a *Saccharomyces cerevisiae* Fermentation Product on the Feedlot Performance and Carcass Merit of Hair Lambs Offered an Annual Ryegrass-Hay-Based Finishing Diet: A Pilot Study

**DOI:** 10.3390/ani13162630

**Published:** 2023-08-15

**Authors:** Justin C. Burt, Jamie A. Boyd, Lisa L. Baxter, Ivan A. Garcia-Galicia, Brittany P. Kerley

**Affiliations:** 1 Department of Crop and Soil Science, University of Georgia, Tifton, GA 31793, USA; 2 Department of Animal Science, Sul Ross State University, Alpine, TX 79830, USA; 3 Facultad de Zootecnia y Ecología, Universidad Autonoma de Chihuahua, Chihuahua 31453, Mexico; 4 Department of Veterinary Science, University of Kentucky, Lexington, KY 40506, USA

**Keywords:** hair lambs, *S. cerevisiae*, fermentation product, feedlot, annual ryegrass hay

## Abstract

**Simple Summary:**

Production of small ruminants, specifically hair sheep, has increased in recent years and has resulted in an increased presence of hair sheep in feedlots for finishing. In 2017, the United States banned the use of antibiotic growth promoters. This ban has resulted in an interest in non-medicated feed additives, such as yeast, to aid in growth promotion. While hair lambs tend to take longer to reach an adequate finishing weight, the utilization of a yeast fermentation product may aid as a natural growth promoter in the feedlot. Additionally, interest in grass-based finishing systems has grown, but there are limited data on using stored cool-season forages as the base of the diet. Based on the results of this experiment, the inclusion of a yeast fermentation product can improve the feedlot performance and carcass texture of hair lambs in a feedlot with an annual ryegrass-hay-based diet.

**Abstract:**

Hair sheep production has increased in recent years, which has resulted in an increased presence in feedlots. Additionally, grass-based finishing systems for ruminant animal production have increased. Data are limited for finishing hair lambs on diets based on cool-season hay. The objective was to evaluate a *Saccharomyces cerevisiae* fermentation product (SCFP) on the feedlot performance and carcass characteristics of Katahdin lambs offered an annual ryegrass (*Lolium multiflorum*)-hay-based diet. Twenty-four Katahdin lambs (21.5 ± 2.5 kg BW) were assigned to either the control (CON) or the yeast-supplemented group (SCFP) in a completely randomized design. Lambs were offered a 14% crude protein total mixed ration diet based on annual ryegrass hay. The SCFP group also received the yeast supplement in their diet. Lambs in the SCFP group had a higher final body weight (*p* < 0.01) and ADG (*p* = 0.01). Less maximum and total energy were required to cut SCFP lamb meat compared to CON lamb meat (*p* < 0.03). Results from this study indicated that SCFP supplementation may prove to be beneficial in hair lamb finishing diets. Future research will need to specifically evaluate the use of these products in hair lambs with a larger sample size.

## 1. Introduction

The use of growth-promoting antibiotics in animal feeds has been banned in the United States since 2017 [1], which has led to an increased interest in using non-medicated feed additives as alternatives for improving the growth of livestock in feedlots. Several feed additives have been shown to improve animal gains and feed efficiency and reduce animal morbidity [2]. These products often include ionophores, exogenous enzymes, direct-fed microbials, yeasts, and other prebiotic and probiotic supplements in the diet [2].

Yeast products have been commonly used in the diets of production animals [3]. Supplementation of yeast as a feed additive, such as *Saccharomyces cerevisiae* or *Saccharomyces cerevisiae* fermentation products, is typically used in ruminant production animal diets to enhance the nutritional digestibility of low nutritive value forages [4,5], feed intake [6,7,8], average daily gain (ADG) [7,9,10,11], and carcass characteristics [12,13,14,15]. *Saccharomyces cerevisiae* fermentation product (SCFP) is a product of yeast fermentation that is different from live yeast. *Saccharomyces cerevisiae* fermentation products are produced in an anaerobic fermentation process by fermenting a liquid (i.e., cane molasses), roughage products, and processed grain by-products with *Saccharomyces cerevisiae* [16,17,18]. These products have been shown to improve ruminal fermentation and fiber digestion in a cost-effective manner [19].

In recent years, consumer demand has shifted towards more grass-finished meat products compared to the typical grain-finished product [20]. Traditionally, animals that are finished on a concentrated diet finish faster than grass-finished animals; however, grass-finished animals have leaner carcasses by comparison to concentrate finished animals [21,22,23,24,25,26]. One forage that can be utilized in these forage finishing systems is annual ryegrass (*Lolium multiflorum*). Annual ryegrass is a cool-season annual forage that is well known for its high nutritive value and has been shown to improve animal growth and performance when grazed [27]. While commonly used in grazing, limited data currently exist on the feeding of annual ryegrass hay in feedlot conditions.

Finally, hair sheep have grown in popularity for many small ruminant producers in the United States. Their popularity, primarily in the eastern United States, stems from their low-maintenance and exceptional mothering ability coupled with increased parasite resistance compared to wool sheep [28,29]. These traits can make hair sheep a valuable livestock species in the semi-arid regions of the US as well, including West Texas. This increase in popularity increased the number of hair sheep entering feedlots for finishing [30]. The fattening of lambs in feedlot finishing systems is generally based on grain concentrate and hay-based diets [31,32]. However, the research is limited regarding the performance of hair sheep under feedlot conditions, specifically, when offered a high nutritive value hay-based diet instead of a high concentrate-based diet [30,31,32].

Therefore, the objective of this study was to determine if supplementation of a *Saccharomyces cerevisiae* fermentation product had an impact on the animal performance and meat quality characteristics of Katahdin lambs offered an annual ryegrass-hay-based diet in a feedlot in the Trans-Pecos region of Texas.

## 2. Materials and Methods

This study was conducted from June–October of 2018 at Sul Ross State University—Alpine Campus Range Animal Science Center (30°21′ N, 103°39′ W; elevation 1368 m). All animal care procedures were approved by Sul Ross State University Institutional Animal Care and Use Committee (IACUC AUP# 2018-ALP-AS-101).

### 2.1. Feedlot Study

Twenty-four Katahdin lambs (ewe n = 16; wether n = 8), with an initial BW of 21.6 ± 2.5 kg, were randomly assigned to dietary treatments of either the control diet (CON) or the yeast fermentation product supplemented diet (SCFP). Lambs were blocked by body weight (BW) and sex. All lambs were offered a diet consisting of annual ryegrass hay, a non-medicated commercial sheep pellet (Angelo Pellets, Inc., San Angelo, TX, USA), bermudagrass (*Cynodon dactylon*) hay, wheat (*Triticum aestivum*) straw, and were top-dressed with ground corn (Table 1). The SCFP group received the same CON diet plus supplementation of a yeast fermentation product (Diamond V NaturSafe, Diamond V, Cedar Rapids, IA, USA) at the rate of 4 g hd^−1^ d^−1^ (approximately 0.5% of the diet, which is the manufacturer-recommended dosage). The product was mixed in with ground corn as the carrier. All lambs were pen-fed using a bunk feeder with ad libitum access to feed and water for 100 days. Temperature and humidity data were also collected (Hobo, Onset Computer Corporation, Bourne, MA, USA). The average temperature and relative humidity during the evaluation were 24.7 °C and 56.5%, respectively.

Diet and ingredient samples were collected twice per week and composited by week for chemical analysis. The DM content of the samples was determined by drying in a forced-air oven at 55 °C for 48 h. Samples were ground to pass through a 1 mm Wiley Mill screen (Thomas Scientific, Philadelphia, PA, USA) for chemical analysis of DM [33], NDF and ADF [34], CP (Kjeltec 2200, Seattle, WA, USA), and ether extract [33].

### 2.2. Carcass Trait Determination

After the termination of the feeding experiment, wether lambs (n = 7; CON = 3, SCFP = 4) were sent to the Sul Ross State University Meat Lab to be harvested using standard commercial procedures and in accordance with Texas State inspection [35]. Only wether lambs were utilized for carcass evaluations because the ewe lambs were being utilized as breeding stock. Prior to slaughter, lambs were fasted for 24 h with ad libitum access to water. Lamb live weights were collected pre-slaughter, and hot carcass weights (HCW) were collected post-slaughter. Liver and ruminal wall samples were collected from each carcass and visually analyzed for any abscesses and signs of ruminal acidosis, respectively. After exsanguination, carcasses were sprayed with lactic acid before being placed in the chill cooler for 24 h. At 24 h post-mortem, approximately 450 g samples from the Longissimus muscle were excised from both halves of each lamb carcass (up to the 12th and/or 13th rib). The samples were vacuum-packed individually by animal and stored in a −80 °C freezer for later analysis at the Universidad Autónoma de Chihuahua, Chihuahua, Mexico.

Longissimus muscle samples were cut into steaks (2 cm thickness), and every steak was used to analyze color, fatty acid profile [36,37], lipid oxidation [38], and shear force [39]. The samples were not aged for longer than 2 d (the time that it took to perform the analysis in Mexico). Surface color of all muscles was measured in triplicate at three positions on every steak with a Minolta Chromameter (Konica Minolta Camera, U.K. Aperture, 8 mm. Illuminant C, D65. Standard observer, C: Y = 94.2, x = 0.3130 and y = 0.3190). Following the CIE Lab methodology [40], L* (lightness), a* (redness), and b* (yellowness) coordinates were measured. Fat extraction was performed using the method described by Bligh and Dyer [36]. Previously, the meat samples were allowed to bloom for at least twenty minutes before analysis. Fatty acid profile was determined by derivatization, saponification, and esterification following the method of AOAC [37]. The concentration of fatty acids was determined by gas chromatography (Clauruss 400. Perkin Helmer Instruments Inc., Waltham, MA, USA) with a polar column of 0.25 mm diameter, 100 m long, and 0.20 µm film (Supelco™ 24110-V SP+M2080, Sigma, Bellfonte, PA, USA). Fatty acid peaks were identified based on retention times according to the standard SupelcoTM 37 Component FAME Mix (Sigma, Bellefonte, PA, USA). Fatty acids are reported as percentage of the total. Lipid oxidation was determined by measuring thiobarbituric acid reactive substances (TBARS) in triplicate [41]. Lipid oxidation reported units are mg of malonaldehyde (MDA)/kg of muscle. Texture was evaluated using Warner–Bratzler shear force (kgf) [39].

### 2.3. Statistical Analysis

One wether was removed from the CON group due to illness, and their data were not included in any of the analyses. Data were analyzed using PROC GLM in SAS v.9.4 [42]. For the feedlot evaluation, the fixed effects were sex and treatment, while in the carcass analysis, the fixed effects were treatment. Block was considered a random effect for both analyses. Differences were considered significant at *p* ≤ 0.05 and tendency at 0.05 < *p* ≤ 0.10.

## 3. Results

### 3.1. Animal Feedlot Evaluation

The feedlot performance metrics are presented in Table 2. There was a difference in the final BW based on the diet of the lambs, where the lambs in the SCFP treatment had a higher final BW compared to the CON group lambs (Table 2; *p* < 0.01). Furthermore, lambs fed SCFP had a higher ADG than the CON treatment (Table 2; *p* < 0.01). No differences were observed for the DMI or feed efficiency based on diet (Table 2; *p* = 0.41 and *p* = 0.40, respectively).

### 3.2. Carcass Merit Evaluation

No difference was observed for the analysis of hot carcass weight (HCW) or dressing percentage (*p* = 0.27 and *p* = 0.22) between the CON and SCFP treatments (Table 3). There were no differences between the CON and SCFP treatments for the color measurements of L* (*p* = 0.85), a* (*p* = 0.87), and b* (*p* = 0.17; Table 3), or for lipid oxidation (*p* = 0.40; Table 3).

Conversely, there was an improvement in the texture when comparing the CON and SCFP groups (Table 3). The maximum amount of energy needed to cut (*p* = 0.03) and the total energy needed to cut the meat samples (*p* < 0.01) were less for the lambs fed SCFP. Additionally, the carcasses from lambs fed SCFP contained less C14:0 fatty acid (*p* = 0.03) compared to the control (Table 4). There was a tendency for these carcasses to have lower C18:1n9t and higher C18:1n9c fatty acids (Table 4; *p* < 0.07) compared to the CON treatment (Table 4).

## 4. Discussion

### 4.1. Feedlot Evaluation

Traditionally, studies involving supplementing with SCFP in ruminant diets were primarily focused on the production of dairy [3] or beef cattle [43], with no data reporting its use in sheep in a feedlot setting. Therefore, the results of this experiment can only be compared to cattle experiments. The differences observed in the finishing BW in the current study are consistent with previous research using an SCFP in dairy calves. Lesmeister et al. found dairy calves supplemented with an SCFP at 1% DM or at 2% DM (*p* < 0.03) had a higher finishing BW versus the control group [44]. Additionally, Lesmeister et al. reported that dairy calves supplemented with an SCFP tended to have a higher ADG than those not receiving the supplement of an SCFP [44]. Schingoethe et al. reported that the supplementation of an SCFP in the diet of dairy cows tended to have an improved feed efficiency [45].

In studies evaluating an SCFP in beef cattle feedlot diets, Geng et al. and Paulus et al. did not report differences in the final BW, ADG, DMI, and feed efficiency of beef cattle receiving an SCFP in comparison to the control groups [46,47]. In a meta-analysis by Wagner et al., it was reported that beef cattle receiving an SCFP had a higher finishing BW, ADG, and DMI compared to the control group not receiving an SCFP [43]. While feed efficiency was not improved in the current study, it is suggested that the inclusion of SCFP in the diet could improve the ruminal conditions, resulting in a greater volatile fatty acid production which in turn influences the overall animal growth and performance [43]. A potential explanation for this could be that the diets being consumed in the beef cattle feedlot were more concentrate based, with none being solely stored forage based [43]. While outside the scope of the current evaluation, future research should evaluate the ruminal condition and the extent to which SCFPs impact the ruminal microbiota communities, specifically in a predominate forage-based diet.

### 4.2. Carcass Merit Evaluation

The diet provided to animals influences the intake and digestibility of nutrients and, as an immediate consequence, the growth performance of animals and their body and carcass compositions [43]. However, there is a paucity of data evaluating the inclusion of an SCFP and the impact it has on the carcass merit of any livestock. The results of HCW in the current evaluation differ from Swyers et al., which reported a reduction in the HCW of beef cattle that were offered an SCFP [48]. The present study showed an improvement in the texture of samples from lambs fed SCFP compared to the CON in that the maximum amount of energy needed to cut and the total energy needed to cut the meat samples was reduced. This was supported by Geng et al., who reported a reduction in shear force for the sirloin steak of SCFP-fed versus control bulls (6.74 ± 0.30 and 8.44 ± 0.30 kg, respectively) [46].

It should also be noted that the fatty acid profile of lambs in the current evaluation varied by each fatty acid measured. The difference observed for C14:0 between the CON, and SCFP treatment is higher than that reported by Pereira et al. [49], with there being a lower presence in the SCFP lambs. While this was the only difference observed in the current evaluation, there was a tendency for both the reduction of C18:1n9t and the increase of the C18:1n9c fatty acids in the lambs offered an SCFP. This is important to note as C18:1n9c is reported to have a positive effect on human health [50]. The improved carcass quality when feeding SCFP could be a result of increased ruminal propionate production, which is converted to glucose in the liver. [35]. The limited sample size of the current study may have impacted the lack of differences observed in the fatty acid profile. Therefore, future work should evaluate these products further with a larger sample size. Research is also needed to determine how an SCFP impacts both concentrate-based and stored-forage-based ruminant finishing systems.

## 5. Conclusions

Results of this study suggest that the inclusion of a *Saccharomyces cerevisiae* fermentation product in an annual ryegrass-hay-based feedlot diet can improve the ADG, final body weight, and feed efficiency of Katahdin lambs. Additionally, the overall texture of the meat was improved when a *Saccharomyces cerevisiae* fermentation product was supplemented into the diet. Future research is warranted with a larger sample size evaluating differing dosages of the *Saccharomyces cerevisiae* fermentation product and how it impacts not only the feedlot performance and carcass merit of hair lambs but how it impacts the ruminal digestibility of various forages in hair lamb diets.

## Figures and Tables

**Table 1:** The average percent dry matter (%DM) of ingredients included in diets and chemical composition of feedlot experimental diets offered to Katahdin ewe and wether lambs under feedlot conditions in Alpine, TX.

		Diet
Ingredient, % of DM	CON ^1^	SCFP ^2,3^
Annual Ryegrass Hay	50.0	50.0
Coastal Bermudagrass Hay	8.6	8.6
Wheat Straw	8.0	8.0
Commercial Sheep Pellet ^4^	29.5	29.5
Ground Corn	3.9	3.9
Chemical Composition, % of DM		
DM	94.0 ± 0.1	93.3 ± 0.1
NDF	33.9 ± 3.1	34.9 ± 3.9
ADF	19.5 ± 1.9	20.0 ± 1.5
CP	18.0 ± 0.2	17.5 ± 0.2
Ether Extract	3.3 ± 0.3	3.2 ± 0.2

^1^ Control Diet (CON) = Annual Ryegrass Hay, Bermudagrass Hay, Wheat Straw, Commercial Sheep Pellet, and Ground Corn. ^2^ Treatment Diet (SCFP) = CON diet + 4 g hd^−1^d^−1^ of *Saccharomyces cerevisiae* fermentation product. ^3^ Diamond V NaturSafe, Diamond V, Cedar Rapids, IA. ^4^ Commercial Sheep Pellet Ingredients: grain products, plant protein products, dehydrated alfalfa meal, roughage product, cane molasses, ammonia chloride, calcium carbonate, salt, sodium selenite, and vitamin A supplement.

**Table 2:** The feedlot performance of Katahdin ewe and weather lambs fed diets with and without a *Saccharomyces cerevisiae* fermentation product under feedlot conditions in Alpine, TX.

	Treatments			
Item	CON ^1^	SCFP ^2^	SEM ^3^	*p*-Value
Initial BW (kg) ^4^	19.5	19.6	0.89	0.93
Final BW (kg)	34.2	36.7	0.38	<0.01
ADG (kg) ^5^	0.15	0.16	0.01	<0.01
DMI (kg DM day^−1^) ^6^	1.20	1.19	0.11	0.41
FeedEff ^7^	0.02	0.01	0.001	0.40

Note: The CON group (n = 11) was comprised of ewe lambs n = 8 and wether lambs n = 3. The SCFP group (n = 12) was comprised of ewe lambs n = 8 and wether lambs n = 4. ^1^ Control Diet (CON) = Annual Ryegrass Hay, Bermudagrass Hay, Wheat Straw, Commercial Sheep Pellet, and Ground Corn. ^2^ Treatment Diet (SCFP) = CON diet + 4 g hd^−1^d^−1^ of *Saccharomyces cerevisiae* fermentation product; Diamond V NaturSafe, Diamond V, Cedar Rapids, IA. ^3^ SEM: Standard error of the mean. ^4^ BW: Body weight. ^5^ ADG: Average daily gain. ^6^ DMI: Dry matter intake. ^7^ FeedEff: Feed Efficiency = Average Daily Gain ÷ Dry Matter Intake. Significance was defined as *p* ≤ 0.05 and a tendency at 0.05 < *p* ≤ 0.10.

**Table 3:** Hot carcass weight and dressing percentage of Katahdin wether lambs supplemented with and without a *Saccharomyces cerevisiae* fermentation product.

	Treatment			
Item	CON ^1^	SCFP ^2^	SEM ^3^	*p*-Value
HCW ^4^ (kg)	13.3	16.6	1.56	0.27
Dress percentage (%)	42.7	45.6	1.67	0.22
Max energy to cut (kgf)	4.1	3.1	0.31	0.03
Total energy to cut (kgf)	17.5	12.5	1.26	<0.01
Lipid oxidation (mg MDA^-^kg^−1^ muscle) ^5^	1.2	1.6	0.29	0.40
Color: L*	45.2	45.1	0.41	0.85
Color: a*	11.6	11.6	0.25	0.87
Color: b*	8.6	8.4	0.11	0.17

Note: Meat samples were taken from wether lambs only (n = 7; CON = 3, SCFP = 4). ^1^ Control Diet (CON) = Annual Ryegrass Hay, Bermudagrass Hay, Wheat Straw, Commercial Sheep Pellet, and Ground Corn. ^2^ Treatment Diet (SCFP) = CON diet + 4 g hd^−1^d^−1^ of *Saccharomyces cerevisiae* fermentation product; Diamond V NaturSafe, Diamond V, Cedar Rapids, IA. ^3^ Standard error of the mean. ^4^ HCW: Hot carcass weight. ^5^ MDA: malonaldehyde. Significance was defined as *p* ≤ 0.05 and a tendency at 0.05 < *p* ≤ 0.10.

**Table 4:** Fatty acid profile (% of the total) of Katahdin wether lambs supplemented with and without a *Saccharomyces cerevisiae* fermentation product.

	Treatments			
Fatty Acid	CON ^1^	SCFP ^2^	SEM ^3^	*p*-Value
C14:0	2.14	1.91	0.15	0.03
C14:1n5	0.12	0.13	0.03	0.69
C16	26.66	26.09	1.12	0.59
C16:1n7	1.56	0.98	0.50	0.17
C18	14.35	15.02	1.74	0.69
C18:1n9t	3.04	1.96	0.75	0.06
C18:1n9c	44.38	46.94	1.81	0.07
C18:2n6t	0.27	0.24	0.05	0.54
C18:2n6c	3.12	3.11	0.72	1.00
C18:3n6	0.05	0.10	0.07	0.50
C18:3n3	0.04	0.06	0.02	0.42
C20:0	0.35	0.27	0.07	0.13
C20:1n9	0.68	0.54	0.12	0.21
C20:3n3	0.07	0.12	0.04	0.22
C20:4n6	0.13	0.16	0.06	0.55
C20:5n3	1.23	0.48	0.76	0.27
C22:5n3	1.35	1.19	0.27	0.53
C22:6n3	0.21	0.39	0.24	0.40
C24:1n9	0.25	0.33	0.08	0.24
SFA	43.50	43.28	1.56	0.89
MUFA	50.03	50.87	1.19	0.45
PUFA	6.48	5.85	1.22	0.59
n6	3.57	3.62	0.72	0.94
n3	2.91	2.23	0.89	0.41

Note: Meat samples were taken from wether lambs only (n = 7; CON = 3, SCFP = 4). Abbreviations: SFA, saturated fatty acid; MUFA, monosaturated fatty acid; PUFA, polyunsaturated fatty acid; n6, omega-6 fatty acid; n3, omega-3 fatty acid. ^1^ Control Diet (CON) = Annual Ryegrass Hay, Bermudagrass Hay, Wheat Straw, Commercial Sheep Pellet, and Ground Corn. ^2^ Treatment Diet (SCFP) = CON diet + 4 g hd^−1^d^−1^ of *Saccharomyces cerevisiae* fermentation product; Diamond V NaturSafe, Diamond V, Cedar Rapids, IA. ^3^ Standard error of the mean. Significance was defined as *p* ≤ 0.05 and a tendency at 0.05 < *p* ≤ 0.10.

## Data Availability

Not Applicable.
